# Targeted next-generation sequencing of colorectal cancer identified metastatic specific genetic alterations

**DOI:** 10.1186/1753-6561-6-S6-P3

**Published:** 2012-10-01

**Authors:** A Rose Brannon, Efsevia Vakiani, Sasinya Scott, Brooke Sylvester, Krishan Kania, Agnes Viale, David Solit, Michael Berger

**Affiliations:** 1 Department of Pathology, Memorial Sloan Kettering Cancer Center, New York, NY 10065, USA; 2 Human Oncology and Pathogenesis Program, Memorial Sloan Kettering Cancer Center, New York, NY 10065, USA; 3 Genomics Core Laboratory, Memorial Sloan Kettering Cancer Center, New York, NY 10065, USA

## Background

In colorectal cancer (CRC), the third most common cause of cancer and cancer death for both men and women in the USA, the steps necessary for carcinogenesis have been well defined. However, the genetic changes necessary for tumor evolution to metastasis are not as well elucidated. Therefore, we searched for the presence of mutations in key cancer genes in primary and metastatic colorectal cancer to determine whether primary or metastatic tissue is ideal for biomarker analysis and to identify putative biomarkers.

## Materials and methods

Fifty patient-matched primary tumor, metastatic tumor and normal samples from CRC patients were sequenced using a panel of 230 genes that are actionable, have been shown to be mutated in human tumors, and/or are part of commonly dysregulated pathways. This assay, coined IMPACT (Integrated Mutation Profiling of Actionable Cancer Targets), provided sequence data to a median depth of coverage of 644X, allowing for identification of low-frequency genetic events. Mutations, insertion/deletions (indels) and copy number events were analyzed comparing both primary and metastatic samples with the corresponding patient normal.

## Results

The majority of genetic events were identical between primary and metastatic tumors; most frequently mutated were the genes commonly associated with CRC, such as *APC*, *TP53*, *KRAS* and *PIK3CA.* A number of mutations were found only in the primary samples, suggesting that these events were not important for tumor progression. Most interesting were the identification of genetic events specific to the metastatic sample: the majority of these alterations were members of two important proliferative pathways, further lending credence that they might play an important role in metastatic progression. Importantly, one of these pathways can be targeted by a number of FDA-approved therapies.

## Conclusions

While the majority of primary and metastatic tumors present with the same genetic events in key cancer genes, additional events in critical pathways were specific to the corresponding metastatic tumors. Further analysis is in process to determine whether these differences are caused by sampling biases within the primary tumor that could be rectified in clinical analysis. The identification of these genetic variations between primary and metastatic tumors could have significant impact on biomarker analysis as well treatment decisions for CRC patients.

